# The Role of Methionine Aminopeptidase 2 in Lymphangiogenesis

**DOI:** 10.3390/ijms21145148

**Published:** 2020-07-21

**Authors:** Rawnaq Esa, Eliana Steinberg, Dvir Dror, Ouri Schwob, Mehrdad Khajavi, Miriam Maoz, Yael Kinarty, Adi Inbal, Aviad Zick, Ofra Benny

**Affiliations:** 1 The Institute for Drug Research, The School of Pharmacy, Faculty of Medicine, The Hebrew University of Jerusalem, Jerusalem 91120, Israel; Rawnaq.esa@mail.huji.ac.il (R.E.); Eliana.steinberg@mail.huji.ac.il (E.S.); Dvir.dror@mail.huji.ac.il (D.D.); ouris@ekmd.huji.ac.il (O.S.); 2 Boston Children’s Hospital, Harvard Medical School, Boston, MA 02115, USA; mehrdad.khajavi@childrens.harvard.edu; 3 Sharett Institute of Oncology, Hebrew University-Hadassah Medical Center, Jerusalem 91120, Israel; miriamm@hadassah.org.il (M.M.); aviadz@hadassah.org.il (A.Z.); 4 Department of Medical Neurobiology, Institute for Medical Research—Israel-Canada, The Hebrew University of Jerusalem, Hadassah Medical School, Jerusalem 9112002, Israel; yael.kinarty@mail.huji.ac.il (Y.K.); adiin@ekmd.huji.ac.il (A.I.)

**Keywords:** MetAp2, angiogenesis, lymphangiogenesis, metastasis, cancer

## Abstract

During the metastasis process, tumor cells invade the blood circulatory system directly from venous capillaries or indirectly via lymphatic vessels. Understanding the relative contribution of each pathway and identifying the molecular targets that affect both processes is critical for reducing cancer spread. Methionine aminopeptidase 2 (MetAp2) is an intracellular enzyme known to modulate angiogenesis. In this study, we investigated the additional role of MetAp2 in lymphangiogenesis. A histological staining of tumors from human breast-cancer donors was performed in order to detect the level and the localization of MetAp2 and lymphatic capillaries. The basal enzymatic level and activity in vascular and lymphatic endothelial cells were compared, followed by loss of function studies determining the role of MetAp2 in lymphangiogenesis in vitro and in vivo. The results from the histological analyses of the tumor tissues revealed a high MetAp2 expression, with detectable sites of co-localization with lymphatic capillaries. We showed slightly reduced levels of the MetAp2 enzyme and MetAp2 mRNA expression and activity in primary lymphatic cells when compared to the vascular endothelial cells. The genetic and biochemical manipulation of MetAp2 confirmed the dual activity of the enzyme in both vascular and lymphatic remodulation in cell function assays and in a zebrafish model. We found that cancer-related lymphangiogenesis is inhibited in murine models following MetAp2 inhibition treatment. Taken together, our study provides an indication that MetAp2 is a significant contributor to lymphangiogenesis and carries a dual role in both vascular and lymphatic capillary formation. Our data suggests that MetAp2 inhibitors can be effectively used as anti-metastatic broad-spectrum drugs.

## 1. Introduction

Cancer metastasis is associated with a high mortality rate and is the primary cause of cancer morbidity. The spread of tumor cells from their primary origin occurs mainly via blood and lymphatic vessels [1]. The 3-dimensional (3D) fast growth of tumors creates a dynamic microenvironment with elevated demands for nutrients and oxygen exchange as well as metabolic waste disposal which are accommodated by the expedited angiogenesis process [2,3].

Angiogenesis is regulated by a dynamic balance between inducer and inhibitor molecules. Tumor angiogenesis is a multistep process triggered by diverse factors secreted by the hypoxic tumor as well as by the host cells [4]. This process is characterized by a formation of new blood vessels from pre-existing vessels [2,3].

Similar to angiogenesis, the growth and formation of new lymphatic vessels, known as lymphangiogenesis, occurs in response to multiple triggers [5]. Lymphangiogenesis was shown to play a critical role in tumor progression and metastasis [6]. Unlike hematologic vessels, lymphatic vessels are thin and consist of discontinuous membranes that enable the penetration of tumor cells into the lymphatic circulation and the adjacent capillary lumens. In response to secreted signals, both lymphatic and vascular endothelial cells proliferate and migrate toward the stimulus, assembling into tube-like structures [1].

Several pathways were identified as promoting angiogenesis and lymphangiogenesis, the most studied and dominant one being the vascular endothelial growth factor (VEGF) pathway. VEGF, which is overexpressed in most cancers and functions as a crucial regulator in angiogenesis and lymphangiogenesis [3,7,8,9], has become an important target for inhibiting tumor metastasis. Among the various VEGF family members, VEGF-C and VEGF-D are secreted by the tumor and stromal cells, playing a dominant role in mediating tumor-related lymphangiogenesis [6,10,11,12].

However, there are many additional mediators that are involved in capillary formation and remodeling. Among these mediators is MetAp2, which belongs to a family of post-translational modification enzymes, acting downstream to VEGF. There are two forms of MetAp in eukaryotes: MetAp1 and MetAp2, the latter having received more interest. This is because the induction of Metap2 was shown to mediate cell proliferation and lead to G_1_ arrest when inhibited [13,14,15,16,17], since it affects protein synthesis via the post-translation removal of methionine [18,19,20,21].

Given MetAp2’s major role in protein synthesis in activated vascular endothelial cells, we hypothesize that MetAp2 also affects lymphangiogenesis by mediating the lymphatic endothelium formation via shared mechanisms. In patient-derived breast cancer tissues, we found that MetAp2 is expressed and co-localized with lymphatic vessels. At the cellular level, we found that the basal expressions of MetAp2 and its activities in human lymphatic endothelial cells (LECs) versus vascular endothelial cells (VECs) were comparable, with slightly higher levels in VECs. In functionality assays including cell proliferation, adhesion, and tube formation, LECs showed suppression upon exposure to TNP-470, a MetAp2 inhibitor. In vivo, in a non-cancerous context using the zebrafish developmental model, the specific inhibition of MetAp2 led to a significant reduction in the development of lymphatic vessels. Parachordal cells (PACs), which make up the lymphatic vessels, were observed in only 0.76 out of 3 segments after treatment with 500 µM of the MetAp2 inhibitor. Moreover, in a subcutaneous (S.C.) murine model using B16/F10 melanoma tumors overexpressing VEGF-C, systemic treatment with 30 mg/kg q.o.d induced over a 50% reduction in the tumors’ volume. A histological examination of the tumors suggested an associated dual reduction of angiogenesis and lymphangiogenesis induced by MetAp2 inhibition.

Taken together, our data suggest that MetAp2 is a mediator of both angiogenesis and lymphangiogenesis and that its inhibition may slow cancer progression and metastasis.

## 2. Results

### 2.1. MetAp2 Expression and Activity in LECs and Tissues

**Figure 1:**
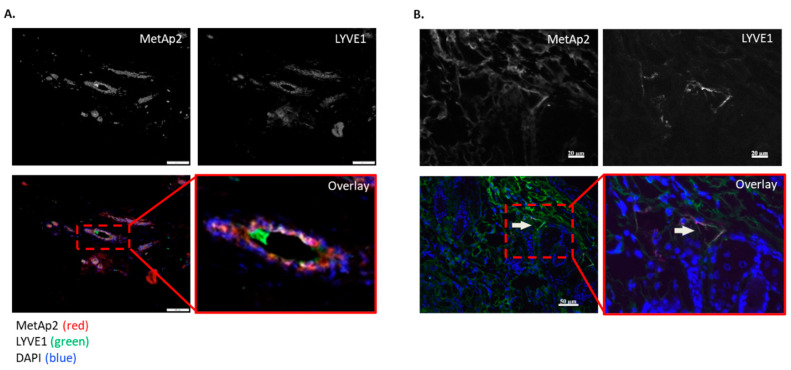
Cancer tissue samples retrieved from two breast cancer patients express MetAp2 and LYVE1. (**A**,**B**) Immunofluorescence staining of MetAp2 (red), LYVE1 (green), and DAPI (blue) reveal lymphatic structures co-localized with MetAp2 expression. Black and white images represent fluorescence in a single channel. Left panel scale bar = 100 µm. Right panel scale bar = 20–50 µm.

Human tumor tissues were surgically resected and collected from breast cancer patients for the histological examination and detection of lymphatic endothelium (indicated by LYVE1 expression) as well as MetAp2 expression. The immunofluorescent staining of the tissue sections showed both MetAp2 and LYVE1 expression with a few loci sites of co-localization (Figure 1A,B).

**Figure 2:**
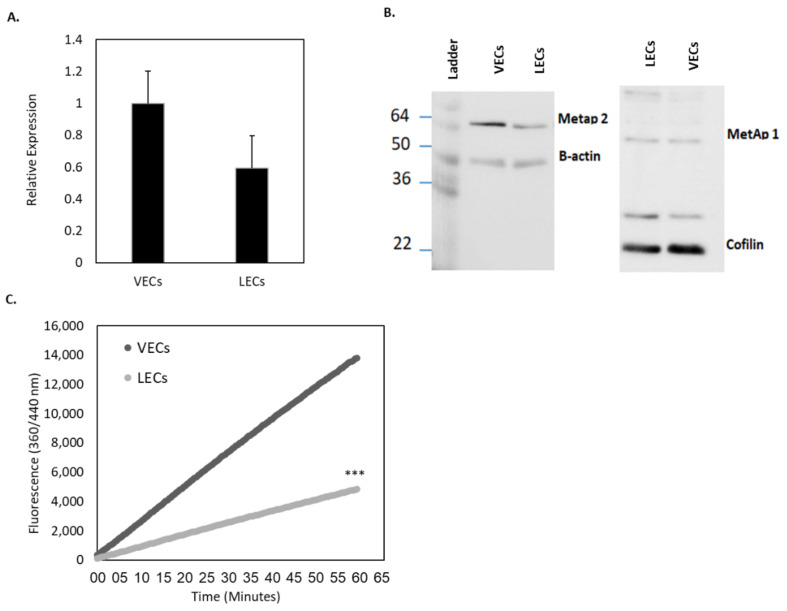
Basal MetAp2 activity and expression in LECs compared with VECs. (**A**) The MetAp2 mRNA levels in LECs (HMVEC-dLyAd cells) is ~60% of the mRNA levels in VECs (HUVECs), as measured by qRT-PCR (5 μg), *n* = 3. (**B**) Western blot analyses for determining the expression of MetAp2 in both cell lines. (**C**) Measurement of the basal activity of MetAp2 in HUVECs and HMVEC-dLyAd cells. A homogenate containing an equal number of cells was added to an L-Met-AMC solution (250 µM). The reaction was monitored for 1 h via the fluorescent quantification of the cleaved substrate. MetAp2 in HMVEC-dLyAd cells showed over a 65% reduction in the enzymatic ability to cleave labeled methionine when compared with HUVECs. The results were normalized to the total cell number. *** *p* < 0.001. The results are presented as mean ± SEM.

Immunoblotting and quantitative real-time PCR (qRT-PCR) were used to measure protein expression at the cell-level. The basal cellular protein levels (baseline expression in the activated state of cells grown in optimal growth conditions) of MetAp2 were comparable in both the vascular and lymphatic endothelial cells (VECs and LECs, respectively), with slightly higher levels of MetAp2 in the vascular endothelium (Figure 2A). In addition, qRT-PCR was used to quantify the MetAp2 mRNA levels in human umbilical vein endothelial cells (HUVECs) and in human dermal microvascular endothelial cells (HMVEC-dLyAd), revealing that the lymphatic cells expressed about 60% of the corresponding mRNA levels in HUVECs, as can be seen in Figure 2B (original western blots are shown in Figure S1). Since enzyme activity is not always correlated with the actual protein levels, the enzymatic activity of MetAp2 was also measured in both LECs and VECs. The N-terminal methionine excision activity was compared in both cell lines (Figure 2C), showing a similar trend as with the mRNA levels. The enzymatic ability to cleave labeled methionine peptides’ end was monitored for 1 h and showed a >65% reduction in activity in HMVEC-dLyAd cells when compared with HUVECs.

### 2.2. The Effect of MetAp2 Inhibition on MetAp2 Expression 

**Figure 3:**
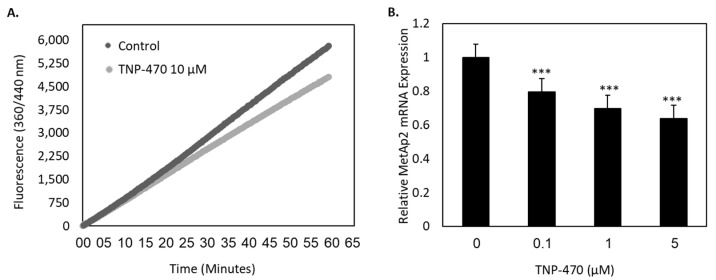
MetAp2 inhibition affects the activity and expression of MetAp2 in LECs. (**A**) The addition of 10 μM TNP-470 treatment inhibits the enzymatic activity of MetAp2 by 23% over the course of 2 h, *n* = 2. (**B**) HMVEC-dLyAd cells were treated with 0, 0.1, 1 and 5 μM of TNP-470, and the MetAp2 mRNA levels were measured after 4 h of incubation using qRT-PCR. The inhibition of MetAp2 reduced the MetAp2 mRNA expression levels to 64%, 70% and 80%, respectively. *n* = 3. *** *p* < 0.001.

In order to inhibit the activity of MetAp2, we used a specific fumagillol analogue, TNP-470, which functions as a MetAp2 antagonist (Figure 3A).

In this assay, we found that 10 μM of TNP-470 led to a ~23% reduction in MetAp2 activity when compared with untreated HMVEC-dLyAd cells. The mRNA levels of MetAp2 were measured after enzyme inhibition in HMVEC-dLyAd cells (Figure 3B). This assay revealed that the treated cells decreased their enzymatic expression to 64%, 70% and 80% when treated with 0.1, 1 and 5 μM TNP-470, respectively, as compared with the untreated HMVEC-dLyAd cells.

### 2.3. MetAp2 Inhibition Reduces LECs Activation In Vitro

**Figure 4:**
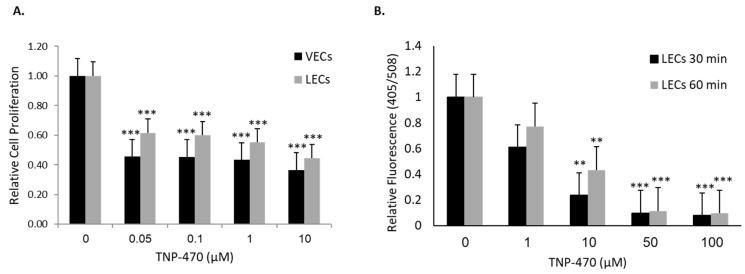
Inhibition of VEC and LEC proliferation and adhesion induced by the inhibition of MetAp2. (**A**) MetAp2 inhibition impairs the ability of both cell lines to proliferate. Cells were treated with different concentrations of TNP-470 for 72 h, after which an MTT assay was conducted to quantify their proliferation. A dose-dependent reduction was observed. *** *p* < 0.005, compared with the control of the same cells. *n* = 5. (**B**) HMVEC-dLyAd cells were stained with DiO and treated with TNP-470; they showed a significant decrease in their ability to adhere. The relative fluorescent cell population was quantified using the Synergy HT multi-mode microplate reader. *n* = 4. ** *p* <0.01, *** *p* < 0.001. The results are presented as mean ± SEM.

Lymphangiogenesis is a multi-step process involving several cellular activities, such as proliferation, adhesion and mobility, which eventually lead to tube formation and tissue remodeling, and which can be assessed individually in specific assays. An MTT assay was conducted to assess the effect of MetAp2 inhibition on lymphatic cell proliferation. HMVEC-dLyAd cells were exposed to different concentrations of TNP-470, ranging from 0 to 10 μM, which resulted in a significant dose-dependent reduction in proliferation following a 72 h incubation period. This MTT assay was also conducted on HUVECs under the same conditions. A reduction of 39–56% in cell proliferation was observed under a 0.05–10 μM TNP-470 treatment, respectively (Figure 4A).

We further measured the effect of MetAp2 inhibition on cell adhesion (Figure 4B). HMVEC-dLyAd cells showed a significant reduction in cell adhesion upon treatment with 10–100 µM of TNP-470. Incubation with 1 µM TNP-470 for 30 and 60 min resulted in a 39% and 29% decrease in the number of adhered cells, respectively. In concentrations of 50- and 100-µM treatments, only a few adherent cells were detected.

**Figure 5:**
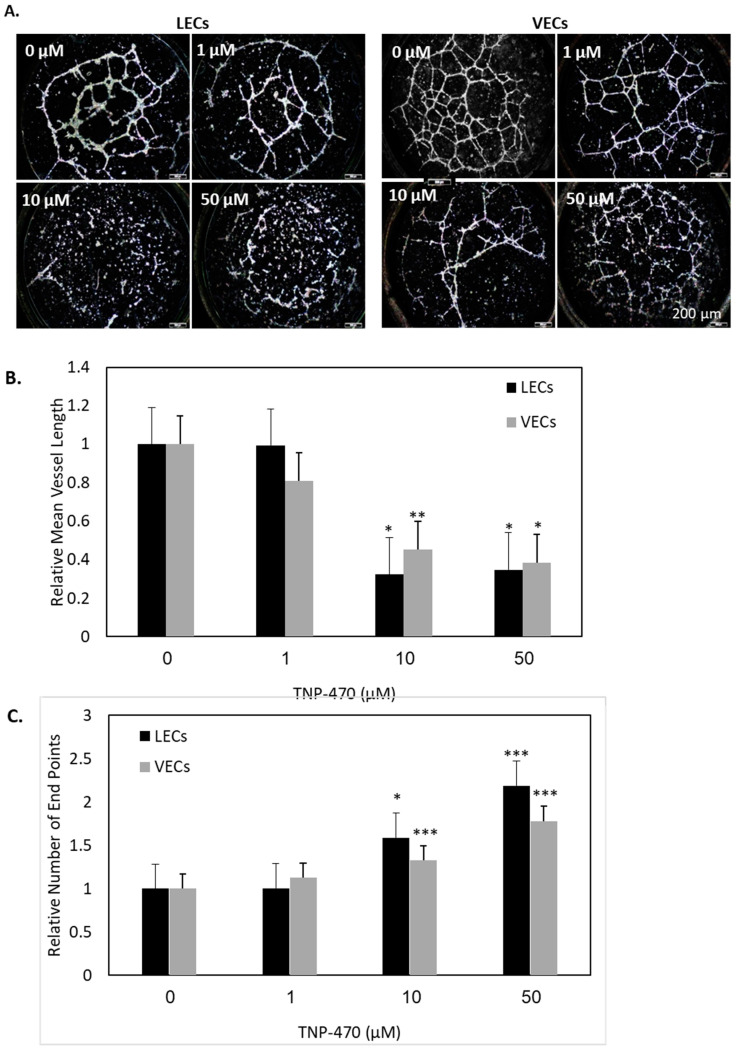
MetAp2 inhibition blocked tube formation in VECs and LECs. (**A**) MetAp2 inhibition impaired HUVECs’ and HMVEC-dLyAd cells’ ability to form tubes. HUVECs and HMVEC-dLyAd cells were seeded on Matrigel and incubated for 14 h under different concentration treatments of TNP-470 (0–50 µM). When no TNP-470 was present, extensive neovascularization was clearly visible and an organized capillary network was formed. When the dose of TNP-470 was elevated to 1–50 μm, the cells’ ability to migrate and reorganize into tubes was impaired. *n* = 8. (**B**,**C**) Metap2 inhibition resulting from exposure to TNP-470 caused a reduction in the relative vessel length and an increase in the relative number of end points in both cell lines, as quantified using the AngioTool image analysis software. *n* = 8. * *p* < 0.05, *** *p* < 0.001. The results are presented as mean ± SEM. Scale bar = 200 μm.

In addition to the monolayer cellular assays, we performed 3D-cellular studies to investigate the effect MetAp2 has on the tube-forming capabilities of HMVEC-dLyAd cells and HUVECs, accomplished through MetAp2 inhibition by TNP-470. Both cell lines were added to Matrigel-coated wells and exposed to different concentrations of TNP-470. Cells treated with the inhibitor showed a significant decrease in the number of meshes and tubes that were formed, and instead formed small foci (Figure 5A).

The 1-µM treatment showed no significant effect on the vessel length. Higher doses of TNP-470, 10 and 50 µM reduced the average vessel length in HMVEC-dLyAd cells by 65% and 69%, respectively (Figure 5B). The total number of end points was 1.6- and 2.1-fold higher than the number of end points of untreated HMVEC-dLyAd cells when subjected to 10 and 50 µM of the inhibitor, respectively. HUVECs showed comparable results but were slightly less susceptible to the treatment (Figure 5C).

### 2.4. In Vivo Anti-Lymphangiogenesis Effects

**Figure 6:**
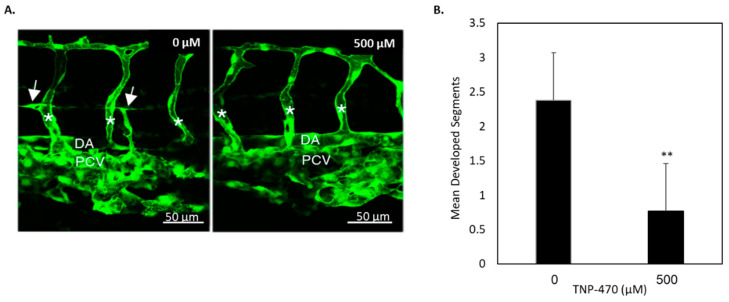
Reduction in the lymphatic vasculature in TNP-470-treated zebrafish embryos carrying the *fli1:EGFP^y1^* transgene. (**A**) Zebrafish treated with 500 μM of TNP-470 showed a reduced lymphatic vasculature (right) when compared with untreated zebrafish (left). Images were acquired using a Zeiss LSM700 confocal microscope and an Axio Imager M2 compound microscope. (**B**) Statistical analysis of the presence of lymphatic vessels of the treated and untreated groups. DA, dorsal aorta; PCV, posterior cardinal vein. Asterisks mark ISVs, and arrows point at PACs. *n* = 17. ** *p* < 0.01. The results are presented as mean ± SEM. Scale bar = 50 μm.

To further investigate whether our in vitro observations also occurred in vivo, we utilized well-established in vivo models of lymphangiogenesis formation. First, we studied the effect of MetAp2 inhibition on the early development of lymphatic vessels using the zebrafish developmental model. Zebrafish embryos carrying the *fli1:EGFP^y1^* transgene were treated 29 hours post-fertilization (hpf) with 500 µM of TNP-470. In these embryos, enhanced green fluorescent protein (EGFP) is expressed in both vascular and lymphatic endothelial cells. To monitor lymphangiogenesis, we examined the presence of PACs, which generate the lymphatic vasculature. Parachordal vessels (PAVs) are present from around 48 hpf, whereas trunk endothelial vasculature, including intersomitic vessels (ISVs), is formed by 29 hpf. Therefore, by adding TNP-470 at 29 hpf we were able to avoid compromising the formation of trunk endothelial vasculature that could have a secondary effect on lymphangiogenesis. At 52 hpf, we imaged a region in the embryos that comprised three segments adjacent to the trunk. The presence of PACs served as a marker of lymphangiogenesis, and the number of segments with PACs was counted. In the control group, PACs were observed in 2.38 out of 3 segments, an average of 79%. In the group treated with 500 µM TNP-470, PACs were observed in only 0.76 out of 3 segments, an average of 25%, showing a statistically significant difference (Figure 6).

**Figure 7:**
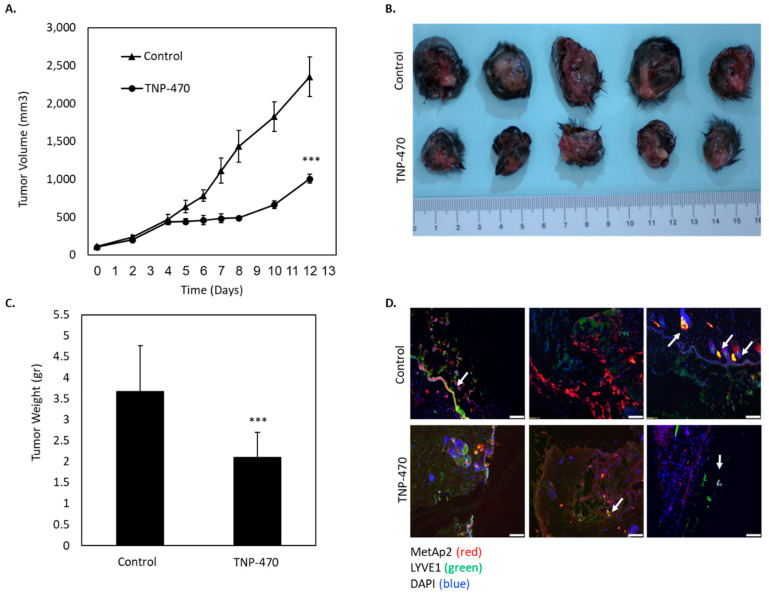
MetAp2 inhibition suppresses the growth of murine melanoma tumors. C57/BL6J mice were injected S.C. with B16/F10 cells, overexpressing VEGF-C (1.5 × 10^6^ cells/tumor). The mice were divided into two groups, untreated and treated with TNP-470 (30 mg/kg q.o.d). *n* = 8–10. (**A**) The tumor volume after 12 days of treatment with TNP-470 was significantly lower (2.3-fold) when compared with the untreated group. *** *p* < 0.005. (**B**) Representative images of tumors extracted from the untreated and treated groups after 12 days of TNP-470 treatment. (**C**) The mean weight of the tumors extracted from the untreated and treated mice following 12 days of TNP-470 treatment. *n* = 8. *** *p* < 0.005. (**D**) Immunofluorescent staining for MetAp2 (red), LYVE1 (green) and DAPI (blue) shows lymphatic structures (white arrows). The results are presented as mean ± SEM. Scale bar = 100 μm.

Finally, we investigated the anti-tumor activity of MetAp2 inhibition in lymphangiogenic-rich tissues. We induced murine melanoma in C57/BL6J mice by S.C. injecting them with 1.5 × 10^6^ B16/F10 cells per tumor, overexpressing VEGF-C. A TNP-470 30 mg/kg intraperitoneal (I.P.) q.o.d treatment was initiated when the tumors reached a size of ~100 mm^3^. After only six days, a significant reduction in the tumor volume was observed in the treated group as compared with the untreated group. On day 12, the tumors in the untreated group were 2.3 times larger in volume than the tumors in the treated group (Figure 7A).

At the end point on day 12, the tumors were weighed and the mean tumor weight of the untreated group was 3.6 g and the treated group had a mean weight of 2.1 g (Figure 7B,C). The immunofluorescent staining of sections obtained from the extracted tumors revealed that MetAp2 and LYVE1 were co-localized and expressed in both the treated and untreated tumors (Figure 7D). However, in the treated samples, co-localization appeared to a lesser extent. In addition, MetAp2^+^LYVE1^+^-expressing cells were more dispersed in the treated tumors, while cells in the untreated samples were organized into vessels. Complete lymphatic vessels were observed more distinctively in the untreated tumors.

The relative cell division rate of B16/F10 cells compared with B16/F10-VEGF-C cells was studied using an MTT assay after 72 h of treatment with 0, 40 and 80 µM of the MetAp2 inhibitor. B16/F10-VEGF-C cells showed a ~50% increase in cell proliferation as compared with B16/F10 cells (Figure S2A). Furthermore, B16/F10-VEGF-C cell proliferation was significantly reduced when treated with 40 and 80 µM as compared with B16/F10 cells (~25–30% reduction), demonstrating the differences between the two models (Figure S2B).

## 3. Discussion

Tumor metastasis is the leading cause of mortality worldwide and is the main cause for cancer-related complications. Most current treatments for invasive cancers are not sufficiently effective [22,23]. Despite the initial promise of anti-angiogenic therapies, the clinical successes of monotherapies have only been partial [3,24].

There is a critical need for complimentary approaches that improve clinical outcomes and lead to higher survival rates in cancer patients. Intervening in the molecular pathways that are involved in lymphangiogenesis may offer a potential therapy target for combating tumor progression and metastasis. While ample research has focused on MetAp2’s involvement in angiogenesis and endothelial cell biology, its role in lymphangiogenesis and in lymphatic endothelial cell biology has not been sufficiently studied.

Here we demonstrated with a series of experiments the effect of MetAp2 inhibition on LECs’ cellular functions and, more specifically, on lymphangiogenesis. Histological samples of human breast cancers showed a high expression of MetAp2 and the co-localization of MetAp2 with lymphatic vasculature (Figure 1). Breast cancer treatment often involves the removal of the axillary lymph nodes where tumor metastasis is usually found [25]. Co-localization may suggest that the lymphatic development is linked with higher MetAp2 levels.

It is well established that MetAp2 is overexpressed in activated endothelium, but its level in primary LECs and its specific role in lymphangiogenesis is yet to be determined [26]. We found that MetAp2 mRNA and the enzyme itself were highly expressed in active LECs (Figure 2). The enzymatic expression and the proteolytic activity were also found to be susceptible to biochemical inhibition by TNP-470 (Figure 3).

After establishing MetAp2’s basal expression and activity, we investigated its effect on cellular functionality in LECs. MetAp2 inhibition in the vascular endothelium is known to regulate cell proliferation through cell cycle arrest in the late G_1_ phase [27]. We found that HMVEC-dLyAd’s cell proliferation was impaired when MetAp2 was inhibited. This was observed when even low concentrations of the antagonist were used. The lowest concentration that was used, 0.05 µM, reduced proliferation by ~50%. These findings indicate the potential antagonistic effects of MetAp2 inhibitors on lymphatic vessel formation. Datta et al. suggested that *fumagillin* and its analogs stabilize MetAp2 in a certain configuration, rather than inhibiting it, which in turn causes an elevation in its affinity for extracellular signal-regulated kinases (ERK1/2). This, consequently, leads to a reduction in MetAp2’s anti-inhibitory protection of eukaryotic initiation factor 2 (eIF2), which in turn decreases the total cellular protein synthesis, induces cell-cycle arrest and impairs the cell’s ability to function [28,29]. This corresponds with our results which showed that treatment with a fumagillin analogue had a relatively small effect on MetAp2 expression in contrast to the cell functionality which was substantially affected. It is probable that the differences in the enzyme levels in VECs, when compared to those in LECs, may explain the higher susceptibility of VECs to enzymatic inhibition. Moreover, the LECs that were used (HMVEC-dLyAd) might contain a small percentage of endothelial cells which, when purified, might show even more significant differences between the two cell lines.

These observations correlate with our in vivo histological analyses which showed that MetAp2 inhibition affected LECs’ remodulation while maintaining relatively steady levels of the MetAp2 enzyme. The immunofluorescence of VEGF-C overexpressing murine melanoma tissue sections showed that in treated tissues, LYVE1+ cells were organized more sporadically and less collectively as vessels as compared with untreated samples. This may be a result of a disruption in the intercellular interactions of lymphatic cells, as confirmed in the tube formation assay, occurring without substantially lowering the MetAp2 levels in these affected cells.

Forming new lymphatic capillaries depends on the cells’ ability to migrate, form cell-cell interactions and adhere to the extracellular matrix (ECM) [30,31]. MetAp2 inhibition resulted in a significant impairment in the cells’ adherence capabilities to the ECM. Assembly of cells into vessels requires cell adhesion and proliferation. These two processes were impaired when MetAp2 was inhibited (Figure 4). In line with this, HMVEC-dLyAd cells’ morphogenesis was remarkably affected, as demonstrated by the tube formation assay (Figure 5). The cells’ ability to interact was notably reduced, as measured by the high number of vessel ends.

To determine whether our in vitro findings are also present in vivo, cancer and non-cancer animal models were utilized to investigate MetAp2’s activity. The zebrafish model provides a convenient way for the real-time visualization of developmental processes, especially the *Tg*(*fli1:EGFP)^y1^* model which enables one to monitor vasculogenesis. In the zebrafish model, which is similar to mammalian development, we found MetAp2 to be highly involved in lymphatic capillary formation and sprouting. While trunk endothelial vessels developed by 29 hpf, PAVs developed at about 48 hpf, creating an ideal window of time for the inhibition of lymphatic vessel formation without the impairment of blood vasculature development. When zebrafish with a developed blood vasculature were treated with a MetAp2 inhibitor, a significant reduction in lymphatic development was observed (Figure 6), implying MetAp2’s significance in embryonic lymphangiogenesis.

Several studies showed that tumor cells overexpressing VEGF-C created a richer lymphangiogenic microenvironment when compared with wild type tumors [6,10]. In order to enhance lymphangiogenesis in tumors, we injected C57/BL6J mice with melanoma B16/F10 cells overexpressing VEGF-C. Treatment with a MetAp2 inhibitor led to a more than 50% decrease in the tumors’ size, associated with reduced lymphatic vascularization (Figure 7). It should be noted that B16/F10-VEGF-C cells showed a significant increase in proliferation when compared with B16/F10 cells, which might explain the first cells’ high sensitivity to the MatAp2 inhibitor (Figure S2).

Taken together, our data demonstrate that MetAp2 has a dual role in blood and lymphatic vascularization, with specific significant effects on cell recruitment and endothelium remodeling. In our previous studies, we showed the preventative effect of an orally available formulated TNP-470 in melanoma liver metastases in an intrasplenic injection model [32]. These significant results were mainly attributed to its anti-angiogenic activity. However, our current observations suggest the involvement of MetAp2 in lymphangiogenesis and the potential to prevent a lymphatic pro-metastatic process by inhibiting MetAp2. This is of particularly high clinical relevance since lymphatic metastasis is common in different cancers and serves as a prognosis indicator of a disease’s aggressiveness. MetAp2 may prove to be an effective target for both angiogenesis and lymphangiogenesis, and its inhibition may be used one day to supplement current methods for cancer treatment.

## 4. Materials and Methods 

### 4.1. Reagents

TNP-470 (O-(Chloroacetylcarbamoyl)fumagillol), also known as AGM-1470, was purchased from MedChem Partners (Lexington, MA, USA).

### 4.2. Human Breast Carcinoma Histological Staining

The study complies with the Declaration of Helsinki. The study protocol was approved by the Institutional Ethics Committee (0346-12, 04-02-2013). Paraffin-embedded sections were prepared and used for immunofluorescence staining. Paraffin-embedded sections were deparaffinized and rehydrated. Antigen retrieval was performed by immersing the samples in TRIS-EDTA buffer and heating in a microwave oven for 15 min. PBS with 0.1% v/v Tween 20 was used to wash the samples, following each step of the procedure. After 15 min of permeabilization with 0.1% Triton, slides were incubated for 1.5 h at room temperature (RT) with a blocking reagent (3% serum in PBS) in order to reduce non-specific antibody (Ab) binding. Sections were incubated overnight at 4 °C with primary Abs in a blocking solution. The primary Abs included: anti-MetAp2 and anti-LYVE1 for lymphatic microvessel (LV) staining. LV and MetAp2 were detected by Alexa Flour^®^ 488 and Alexa Flour® 647 secondary Abs, respectively, and nuclei were detected by DAPI staining. 

### 4.3. Cell Culture

HUVECs (VECs) and HMVEC-dLyAd cells (LECs) were purchased from Lonza (Walkersville, MD, USA). All cells were characterized before use, mycoplasma-free, using an EZ-PCR Mycoplasma Test Kit (Biological Industries), and were used for the experiments up to passage 12. All cells were kept in a humidified incubator at 37 °C with 5% CO_2_. HUVECs were maintained in a specific medium supplemented with the PeproGrow-MacroV kit (ENDO-BM & GS-MacroV, PeproTech) and penicillin/streptomycin. For HMVEC-dLyAd cells, an EGM^TM^-2 MV Microvascular Endothelial Cell Growth Medium (Lonza, Walkersville, MD, USA) supplemented with penicillin/streptomycin was used. For starvation, primary cells were cultivated in their appropriate medium without supplementation. B16/F10 murine melanoma cell lines were obtained from ATTC (Manassas, VA, USA) and cultivated in DMEM supplemented with 10% FCS and penicillin/streptomycin.

### 4.4. Western Blot

Cells were lysed with RIPA buffer in a protease inhibitor cocktail (Sigma, S8820) for 30 min on ice. Lysates were centrifuged, and the supernatant was collected. Protein content was determined according to the BCA Protein Assay kit (Pierce^TM^, Thermo Fisher Scientific, Cambridge, MA, USA). Proteins (15 μg protein) were separated by a 12.5% Tris-glycine SDS-PAGE and transferred onto a Polyvinylidene difluoride membrane (Millipore Corporation, Billerica, MA, USA). Membranes were blocked for 2 h and then incubated with anti-MetAp2 abs (Ab134124, Abcam, Cambridge, UK) overnight at 4 °C in TBST containing 5% BSA. Membranes were washed three times for 5 min in TBST, incubated with a 1:5000 dilution of goat anti-rabbit secondary ab conjugated to horseradish peroxidase for 1 h (Ab97080, Abcam). β-Actin (Ab49900, Abcam) was used as the loading control.

### 4.5. Total RNA/Protein Extraction and Expression Analysis

Total RNA was isolated from each sample using the RNeasy Kit (Qiagen, Valencia, CA, USA). 5 µg of total RNA from each sample was reverse transcribed with the Superscript First-strand cDNA synthesis kit (Invitrogen, Carlsbad, CA, USA) using a random primer. qRT-PCR for MetAp2 (Hs00199152_m1) and 18S rRNA (Hs03003631_g1) was performed in triplicate using TaqMan Gene Expression assays (Life Technologies, Grand Island, NY, USA). The relative level of each RNA sample was calculated using the ∆∆*C*t method normalized to the corresponding 18S rRNA levels, *n* = 3.

### 4.6. Activity Assay

HUVECs and HMVEC-dLyAd cells were subcultivated using trypsin, then centrifuged and counted. To obtain a homogenate containing an equivalent cell number, cells were centrifuged again and re-suspended in an appropriate volume of cold RIPA buffer containing a protease inhibitor cocktail. Cells were disrupted using a probe sonicator on ice (Sonic Ruptor 400, OMNI International). Insoluble cellular components were removed by centrifugation at 15,000 RPM for 10 min at 4 °C. The protein content of the supernatant was determined according to the Bradford protein assay using BSA as the standard [33]. The enzymatic activity was tested on 5 μg of protein using L-Met-AMC (Santa Cruz Biotechnology) as a substrate. The reaction was performed in an assay buffer (pH 7.5) containing 50 mM HEPES, 0.1 mM CoCl2, 100 mM NaCl and 1 mg/mL PEG 6000, in a final volume of 100 μL. Fluorescence was measured every 20 sec for 1 h at 25 °C using a plate reader (Synergy HT Multi-Mode Microplate Reader, BioTek), *n* = 2. 

### 4.7. Activity Assay Following MetAp2 Inhibition

HMVEC-dLyAd cells were grown to 80–90% confluency and were then washed with cold PBS and scraped on ice using a protease inhibitor-free RIPA buffer. A total of three HMVEC-dLyA dishes were scraped using the same buffer, which was transferred from one plate to the other. Insoluble cellular components were removed by centrifugation at 15,000 RPM for 10 min at 4 °C. The protein content of the supernatant was determined according to the Bradford protein assay using BSA as the standard. The enzymatic assay was performed using 5 µg of protein per sample, as described previously. To test the inhibitory effect of TNP-470, samples were incubated with the inhibitor for 15 min at RT before adding the substrate. An increase of fluorescence, due to substrate degradation during the enzymatic assay, was measured every 25 seconds for 1 h at 25 °C using a plate reader, as previously mentioned, *n* = 2.

### 4.8. Proliferation Assay

HUVECs and HMVEC-dLyAd cells seeded in 96-well plates (2000 cells/well) were exposed to a range of TNP-470 concentrations (0–10 μM) for 72 h. After incubation, MTT (Sigma Aldrich, St. Louis City, MO, USA) was added (0.5 mg/mL) into each well for viability detection and incubated at 37 °C and 5% CO_2_ for 3 h. The absorbance was measured at 570 nm using a plate reader, *n* = 5.

### 4.9. Cell Adhesion Assay

HMVEC-dLyAd cells were stained with a lipophilic tracer, DiO (5 μL/1 × 10^6^ cells), and seeded on a gelatin-coated 96-well plate (12,000 cells/well). Cells were treated with TNP-470 at various concentrations (0–100 μM) and incubated for 30 and 60 min. Unadhered cells were removed, and the relative fluorescence of each cell population was quantified using a plate reader, as previously described, *n* = 4.

### 4.10. Tube Formation Assay

The morphogenic potential of HUVECs and HMVEC-dLyAd cells to form capillary-like structures in vitro was evaluated by seeding cells on Matrigel, a basement membrane matrix (Corning, NY, USA). 10 μL of Matrigel was dispensed into each well in a µ-Plate Angiogenesis 96-well (ibidi GmbH Martinsried, Germany) and allowed to polymerize for 1 h at RT. HMVEC-dLyAd cells and HUVECs (12,000 cells/well) were synchronized by starvation in a serum-free medium for 3 h prior to seeding on the polymerized gel. 1.5 h after seeding, TNP-470 in various concentrations (0–50 μM) was added and left to incubate overnight. Images were taken using an inverted fluorescence microscope (Nikon ECLIPSE Ni-E). The mean vessel length and the total number of end points were quantified using the AngioTool image analysis software, *n* = 8.

### 4.11. Animal Models

Animal experiments were approved by the Institutional Animal Care and Use Committee of the Faculty of Medicine of the Hebrew University and followed the guidelines on the use of laboratory animals. 7–8-week-old male C57BL/6J mice were purchased from Harlan. TNP-470 was dissolved in 70% ethanol to obtain a stock solution of 15 mg/mL. Prior to injecting the mice I.P., the stock solution was diluted at 1:5 using sterile saline.

### 4.12. Zebrafish Analysis

*Tg*(*fli1:EGFP)^y1^* zebrafish were used in this study [34]. To block pigmentation, embryos were raised from 22 hpf in the presence of 0.003% N-Phenylthiourea (PTU; Sigma-Aldrich #P7629). 29 hpf embryos were placed in each well of a 24-well plate in 1 mL egg water containing 0.1% DMSO (control) or 500 µM TNP-470. Embryos were kept at 28.5 °C until imaging took place. For imaging, live embryos were anaesthetized using Tricaine mesylate and were mounted in 0.5% low-melting-point agarose (SeaPlaque, Lonza). Images were acquired using a Zeiss LSM700 confocal microscope and an Axio Imager M2 compound microscope equipped with a 40 × 1.0 NA water objective. Images were exported as TIFF files using ZEN 2009 LE software (Zeiss), and figures were assembled using the Adobe Photoshop CS4 software, *n* = 17.

### 4.13. B16/F10-VEGF-C Plasmid Amplification and Cell Transfection

B16/F10 cells were transfected with VEGF-C plasmids as follows: competent *E. coli* (DH5α, Biolab, Jerusalem, Israel) were transformed by heat shock using 1 µg of VEGF-C plasmid (#EX- K2961-M03) or control plasmid (#EX-NEG-M03) (Gene Copoeia Inc., Rockville, MD, USA), respectively. Cells were then transferred to 1 mL of LB medium and incubated for 1 h at 37 °C under constant shaking. The transformed *E. coli* were spread on LB-Agar plates containing Ampicillin (100 µg/mL) for selection. The petri dishes were incubated overnight at 37 °C, and a selected colony of each plasmid was transferred to 5 mL of Ampicillin LB medium for culture, and an amplification step was performed. Bacteria were centrifuged at 5000× *g* for 10 min, and plasmid purification was performed using the GenElute HP plasmid kit (Sigma-Aldrich, St Louis, MO, USA). The DNA concentration and its purity were determined using a microplate reader. Cells were seeded at 200,000 cells/well in a 6-well plate. After 48 h, cells reached a 90% confluency and were then transfected with 4 µg of plasmid using 10 µL/well of Lipofectamine 2000 (Thermo Fisher Scientific, Van Allen Way, Carlsbad, CA, USA). After an additional 24 h, the selection was performed using 3 mg/mL of Gentamycin (G418 Sulfate, Gold Biotechnology, St Louis, MO, USA).

### 4.14. Murine Melanoma Tumors

S.C. tumors were developed in the lower backs of C57BL/6J mice by injecting 1.5 × 10^6^ B16/F10-VEGF-C cells or B16/F10-control cells (transfected with control plasmid) in 100 μL of PBS containing Matrigel (1:10). Tumors were transcutaneously measured every 1–2 days with a digital caliper, and volumes were calculated according to the ellipsoid formula (Length x Width^2^ × 0.52). Mice were weighted and observed daily throughout the experiment. Mice were treated I.P. q.o.d with 30 mg/kg of TNP-470. On day 12, the mice were sacrificed, and the tumors were surgically removed, weighted, frozen in OCT and cryo-sectioned with Leica CM-1950 for an immunofluorescence analysis, *n* = 8–10.

### 4.15. Statistical Analysis

The results are presented as the mean ± SEM. Studies containing two groups were assessed using the unpaired two-tailed Student’s *t*-test. Studies containing more than three groups were compared and analyzed using a one-way analysis of variance (ANOVA), and significant differences were detected using Tuckey’s multiple comparison post-test. Differences were considered statistically significant for *p* < 0.05.

## Supplementary Materials

Supplementary materials can be found at https://www.mdpi.com/1422-0067/21/14/5148/s1.

## References

[1] Stacker S.A., Baldwin M.E., Achen M.G. (2002). The role of tumor lymphangiogenesis in metastatic spread. FASEB J..

[2] Hanahan D., Weinberg R.A. (2011). Hallmarks of cancer: The next generation. Cell.

[3] Jayson G.C., Kerbel R., Ellis L.M., Harris A.L. (2016). Antiangiogenic therapy in oncology: Current status and future directions. Lancet.

[4] Eskens F.A.L.M. (2004). Angiogenesis inhibitors in clinical development; where are we now and where are we going?. Br. J. Cancer.

[5] Al-Rawi M.A.A., Mansel R.E., Jiang W.G. (2005). Molecular and cellular mechanisms of lymphangiogenesis. Eur. J. Surg. Oncol..

[6] Skobe M., Hawighorst T., Jackson D.G., Prevo R., Janes L., Velasco P., Riccardi L., Alitalo K., Claffey K., Detmar M. (2001). Induction of tumor lymphangiogenesis by VEGF-C promotes breast cancer metastasis. Nat. Med..

[7] Shibuya M., Claesson-Welsh L. (2006). Signal transduction by VEGF receptors in regulation of angiogenesis and lymphangiogenesis. Exp. Cell Res..

[8] Lohela M., Bry M., Tammela T., Alitalo K. (2009). VEGFs and receptors involved in angiogenesis versus lymphangiogenesis. Curr. Opin. Cell Biol..

[9] Plate K.H. (2001). From angiogenesis to lymphangiogenesis. Nat. Med..

[10] Karpanen T., Egeblad M., Karkkainen M.J., Kubo H., Ylä-Herttuala S., Alitalo K. (2001). Vascular endothelial growth factor C promotes tumor lymphangiogenesis and intralymphatic tumor growth. Cancer Res..

[11] Stacker S.A., Caesar C., Baldwin M.E., Thornton G.E., Williams R.A., Prevo R., Jackson D.G., Nishikawa S.I., Kubo H., Achen M.G. (2001). VEGF-D promotes the metastatic spread of tumor cells via the lymphatics. Nat. Med..

[12] Mäkinen T., Veikkola T., Mustjoki S., Karpanen T., Catimel B., Nice E.C., Wise L., Mercer A., Kowalski H., Kerjaschki D. (2001). Isolated lymphatic endothelial cells transduce growth, survival and migratory signals via the VEGF-C/D receptor VEGFR-3. EMBO J..

[13] Wang J., Lou P., Henkin J. (2000). Selective inhibition of endothelial cell proliferation by fumagillin is not due to differential expression of methionine aminopeptidases. J. Cell. Biochem..

[14] Datta B. (2000). MAPs and POEP of the roads from prokaryotic to eukaryotic kingdoms. Biochimie.

[15] Wang J., Sheppard G.S., Lou P., Kawai M., BaMaung N., Erickson S.A., Tucker-Garcia L., Park C., Bouska J., Wang Y.C. (2003). Tumor suppression by a rationally designed reversible inhibitor of methionine aminopeptidase-2. Cancer Res..

[16] Zhang Y., Griffith E.C., Sage J., Jacks T., Liu J.O. (2000). Cell cycle inhibition by the anti-angiogenic agent TNP-470 is mediated by p53 and p21WAF1/CIP1. Proc. Natl. Acad. Sci. USA.

[17] Yeh J.R.J., Mohan R., Crews C.M. (2000). The antiangiogenic agent TNP-470 requires p53 and p21(CIP/WAF) for endothelial cell growth arrest. Proc. Natl. Acad. Sci. USA.

[18] Ben-Bassat A., Bauer K., Chang S.Y., Myambo K., Boosman A. (1987). Processing of the initiation methionine from proteins: Properties of the Escherichia coli methionine aminopeptidase and its gene structure. J. Bacteriol..

[19] Li X., Chang Y.H. (1995). Amino-terminal protein processing in Saccharomyces cerevisiae is an essential function that requires two distinct methionine aminopeptidases. Proc. Natl. Acad. Sci. USA.

[20] Ma A.C.H., Fung T.K., Lin R.H.C., Chung M.I.S., Yang D., Ekker S.C., Leung A.Y.H. (2011). Methionine aminopeptidase 2 is required for HSC initiation and proliferation. Blood.

[21] Yeh J.R.J., Ju R., Brdlik C.M., Zhang W., Zhang Y., Matyskiela M.E., Shotwell J.D., Crews C.M. (2006). Targeted gene disruption of methionine aminopeptidase 2 results in an embryonic gastrulation defect and endothelial cell growth arrest. Proc. Natl. Acad. Sci. USA.

[22] Bear H.D., Tang G., Rastogi P., Geyer C.E., Robidoux A., Atkins J.N., Baez-Diaz L., Brufsky A.M., Mehta R.S., Fehrenbacher L. (2012). Bevacizumab added to neoadjuvant chemotherapy for breast cancer. N. Engl. J. Med..

[23] Hodi F.S., Lawrence D., Lezcano C., Wu X., Zhou J., Sasada T., Zeng W., Giobbie-Hurder A., Atkins M.B., Ibrahim N. (2014). Bevacizumab plus ipilimumab in patients with metastatic melanoma. Cancer Immunol. Res..

[24] Vasudev N.S., Reynolds A.R. (2014). Anti-angiogenic therapy for cancer: Current progress, unresolved questions and future directions. Angiogenesis.

[25] Abass M.O., Gismalla M.D.A., Alsheikh A.A., Elhassan M.M.A. (2018). Axillary lymph node dissection for breast cancer: Efficacy and complication in developing countries. J. Glob. Oncol..

[26] Benita Y., Cao Z., Giallourakis C., Li C., Gardet A., Xavier R.J. (2010). Gene enrichment profiles reveal T-cell development, differentiation, and lineage-specific transcription factors including ZBTB25 as a novel NF-AT repressor. Blood.

[27] Wang J., Tucker L.A., Stavropoulos J., Zhang Q., Wang Y.C., Bukofzer G., Niquette A., Meulbroek J.A., Barnes D.M., Shen J. (2008). Correlation of tumor growth suppression and methionine aminopetidase-2 activity blockade using an orally active inhibitor. Proc. Natl. Acad. Sci. USA.

[28] Datta B. (2009). Roles of P67/MetAP2 as a tumor suppressor. Biochim. Biophys. Acta Rev. Cancer.

[29] Datta B., Datta R. (1999). Induction of apoptosis due to lowering the level of eukaryotic initiation factor 2-associated protein, p67, from mammalian cells by antisense approach. Exp. Cell Res..

[30] Jackson C.J., Knop A., Giles I., Jenkins K., Schrieber L. (1994). VLA-2 mediates the interaction of collagen with endothelium during in vitro vascular tube formation. Cell Biol. Int..

[31] Montesano R., Orci L., Vassalli P. (1983). In vitro rapid organization of endothelial cells into capillary-like networks is promoted by collagen matrices. J. Cell Biol..

[32] Benny O., Fainaru O., Adini A., Cassiola F., Bazinet L., Adini I., Pravda E., Nahmias Y., Koirala S., Corfas G. (2008). An orally delivered small-molecule formulation with antiangiogenic and anticancer activity. Nat. Biotechnol..

[33] Kruger N.J. (1994). The Bradford method for protein quantitation. Methods Mol. Biol..

[34] Lawson N.D., Weinstein B.M. (2002). In vivo imaging of embryonic vascular development using transgenic zebrafish. Dev. Biol..

