# Supplementary material for: The Role of Methionine Aminopeptidase 2 in Lymphangiogenesis
Source: Int J Mol Sci. 2020 Jul 21;21(14):5148. doi: 10.3390/ijms21145148 (PMC7403956; doi:10.3390/ijms21145148)
Supplement: Supplementary file 1 [file ijms-21-05148-s001.pdf]

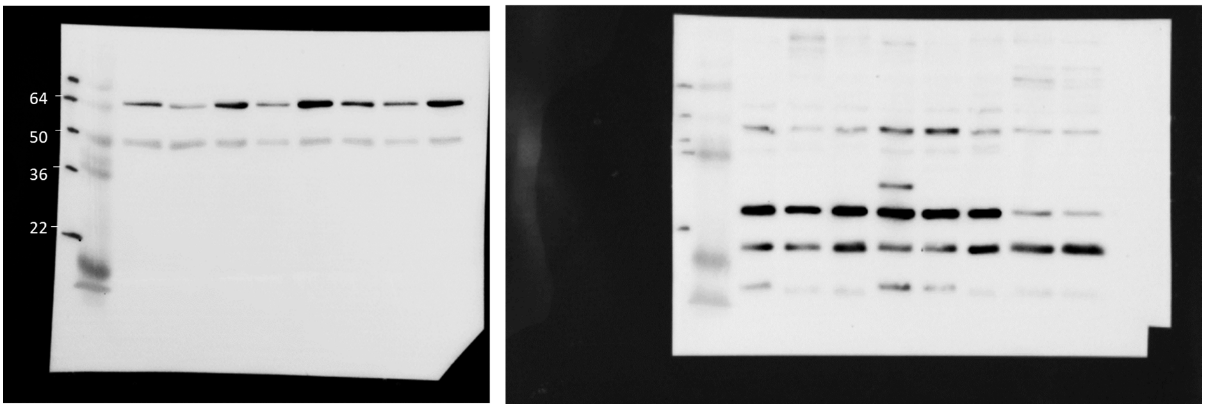

Figure S1: Basal MetAp2 expression in LECs compared with VECs. Western blot analyses for determining the expression of MetAp2 in HUVECs (VECs) and HMVEC-dLyAd (LECs) cells. qRT-PCR was used to quantify the MetAp2 mRNA levels in both cell lines revealing that the lymphatic cells expressed about 60% of the corresponding mRNA levels in VECs

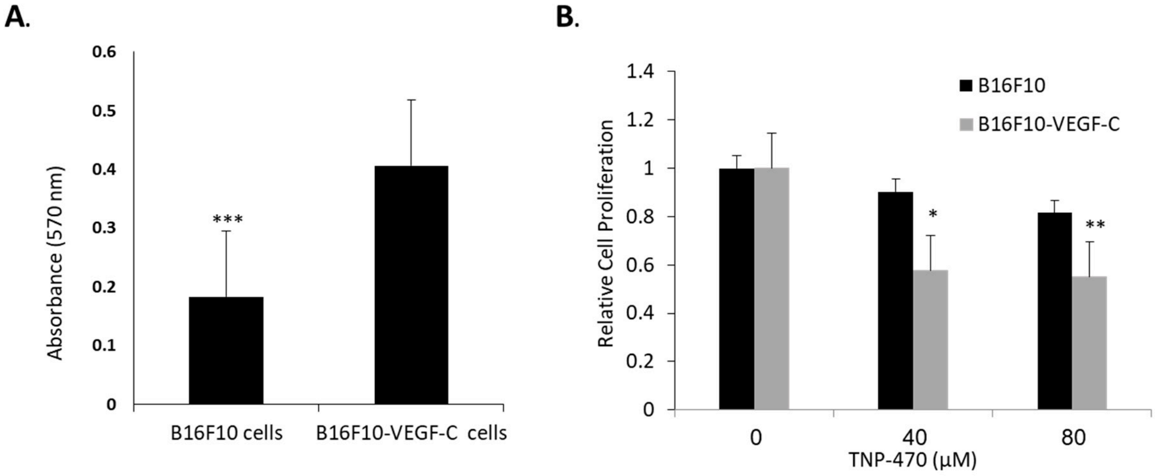

Figure S2: Relative cell division rate of B16F10 and B16F10-VEGF-C cells. (**A**) B16F10 and B16F10-VEGF-C cell proliferation was investigated after 72 h of incubation using an MTT assay. B16F10-VEGF-C cells show ~50% increase in cell proliferation compared with B16F10 cells. n=7. ***p<0.005. (**B**) Cells were treated with 0, 40 and 80 μM of TNP-470 for 72 h after which an MTT assay was conducted to quantify their relative proliferation. B16F10-VEGF-C cell proliferation is significantly reduced with 40 and 80 μM treatment of TNP-470 compared with B16F10 cells (~25-30% reduction). n=6-7. *P<0.05, **P<0.01. Results are presented as mean ± SEM.
